# The patients‘ perspective - a qualitative analysis of experiencing a fracture-related infection

**DOI:** 10.3389/fpsyg.2023.1126826

**Published:** 2023-06-01

**Authors:** Bravena Wimalan, Markus Rupp, Volker Alt, Nike Walter

**Affiliations:** ^1^Department for Trauma Surgery, University Medical Center Regensburg, Regensburg, Germany; ^2^Department for Psychosomatic Medicine, University Medical Center Regensburg, Regensburg, Germany

**Keywords:** qualitative analysis, fracture-related infection, emotional impact, psychological resources, multidisciplinary management

## Abstract

**Introduction:**

Fracture-related infection is a devastating complication confronting the patient with several challenges. To improve the management and to enhance the patients’ wellbeing the focus of this study was to understand the emotional impact and patients’ experiences during the process to identify challenges, difficulties, and resources. For this, a qualitative content analysis of semi-structured interviews according to Graneheim and Lundman was performed.

**Methods:**

In total *n* = 20 patients of a German university orthopedic trauma centre specialized in bone and joint infections were recruited using a purposive sampling strategy. The patients were treated at the hospital between 2019 and 2021 and underwent at least one surgery. Individual in-person interviews were performed by one researcher based on a semi-structured guide, which was previously conceptualized. Content analysis according to Graneheim and Lundman was performed on the transcripts by two of the researchers independently.

**Results:**

The following major themes emerged: (i) the emotional and mental aspects highlighting the fact that FRI patients faced severe restrictions in their day-to-day life, which resulted in dependency on others and frustration, as well as future concerns showing that patients could not overcome a state of anxiety and fear even after successful treatment, (ii) socioeconomic consequences confronting patients with consequences on the job and in finances where they often feel helpless, and (iii) resources emphasizing the role of spirituality as a coping strategy and yoga exercises for keeping the positivity.

**Conclusion:**

This study emphasized the challenge of fracture-related infection management and associated consequences from the patients’ perspective. Not being well informed about possible negative outcomes or restrictions makes it harder for patients to accept the situation and patients expressed a need for better information and certainty. Also, patients developed constant anxiety and other psychological disturbances, highlighting the potential benefit of psychological support and patient-peer support to exchange experiences.

## Introduction

In trauma surgery, reduction and internal fixation are applied to restore skeletal integrity. The use of an intramedullary ivory peg for the treatment of complicated fractures to restore skeletal integrity was reported as early as 1886 (Bekos et al., 2021). Since then, reduction and internal fixation techniques have been advanced, which are nowadays an integral part of orthopedic surgery. Over the last years, the incidence of fractures increased, especially in older adults (Rupp et al., 2021). One of the major complications after fracture fixation utilizing is implant-related infection. Rates of developing a posttraumatic infection are reported to be around 1–2% for closed fractures ranging up to exceeding 30% for Gustilo-Anderson type III open tibia fractures (Trampuz and Zimmerli, 2006; Ktistakis et al., 2014).

In light of increasing numbers of fractures, especially in older adults incidence of fracture-related infections (FRI) can be expected to rise as well (Walter et al., 2021b). The management of FRI is challenging. Depending on several factors, often multiple staged surgeries are needed for the eradication of infection and finally bony consolidation (Metsemakers et al., 2018a). Success rates only vary between 70–90% with a recurrence of the disease in 6–9% of the patients. Several limitations such as immobility up to amputations of the affected limb, prolonged length of stay in hospital, multiple surgeries, side effects of antibiotic medication, and further socioeconomic issues can occur despite a variety of treatment concepts (Bose et al., 2015; Alt and Giannoudis, 2019; Bezstarosti et al., 2019).

However, success in the treatment of FRIs is mainly determined by infection eradication and there are very few data in the literature focusing on patient-reported outcomes. Some studies provided insights in the detrimental effect on patients’ quality of life (Johnson et al., 2019). For instance, it has been shown that even years after successful treatment of FRI of the long bones in terms of eradication of infection and stable bone consolidation patients do not reach scores of a normative reference population (Walter et al., 2021a). Recently, a few quantitative analyses capturing patients’ experiences were brought into the focus of orthopedic and trauma surgery research (Moore et al., 2015; Palmer et al., 2020). Especially, patients’ experience and the emotional impact following one- and two-stage revision for periprosthetic joint infection (PJI) and the need for physical and psychological support during the treatment period was highlighted by conducting in-depth qualitative research interviews (Moore et al., 2015). In addition, the surgeons’ challenge of making treatment decisions has been addressed (Mallon et al., 2018; Palmer et al., 2020). Also, in the case of FRI treatment, the complexity of factors to be considered for optimal management can sometimes surpass the experience of even skilled orthopedic and trauma surgeons. Thus, it is crucial to involve multidisciplinary specialists and to consider the patient’s psychological response to the trauma and the subsequent infectious complication (Rupp et al., 2023).

However, hitherto the experience of undergoing management for an FRI has not been elucidated yet from the patients’ perspective. Therefore, to improve the management and to enhance the patients’ wellbeing the focus of this study was to understand the emotional impact and patients’ experiences during the process to identify challenges, difficulties, and resources. For this, a qualitative content analysis of semi-structured interviews according to Graneheim and Lundman was performed.

## Materials and methods

The study population was identified using a purposive sampling strategy (Coyne, 1997; Palinkas et al., 2015). Patients treated in a trauma centre in Germany between 2019 and 2021 were identified by the international classification of disease (ICD) 10 diagnosis “T84.6 infection and inflammatory reaction due to internal fixation device.” Following the 2018 international consensus meeting on musculoskeletal infection(Metsemakers et al., 2018b), FRI was confirmed by the presence of at least one of the following confirmatory criteria: (1) fistula, sinus tract or wound breakdown, (2) purulent drainage or presence of pus during surgery, (3) phenotypically indistinguishable organisms identified by culture from at least two separate deep tissue/implant specimens (including sonication fluid), and (4) histopathological findings (presence of microorganisms in deep tissue specimens or presence of > five PMN/HPF). Besides confirmed FRI, only patients with at least one surgical treatment were included, whereby patients with multiple fractures were excluded.

Participants were approached by telephone and asked for their study participation after explaining the background and the purpose of the interviews. In total *n* = 31 patients were approached, whereby = 7 were not reachable and *n* = 4 declined their participation. Informed consent was obtained from all participants prior to the interviews. The study was approved by the institutional ethics committee of the University Hospital Regensburg according to the Helsinki Convention (file number 21–2464-101).

A semi-structured interview guide was conceptualized by the authors and pilot tested. In-depth qualitative research interviews were conducted in person between March 2021 and October 2021 by an independent female researcher with a background in psychosomatic medicine in an undisturbed office room. The interviewer was occupied at the same clinic, however, no relationship with the participant was established prior to the study commencement. Interviews lasted between 28:56 and 46:22 min. No repeat interviews were carried out and no field notes were made during the interview. The following areas were covered in the interviews: experiences of the time in the hospital, challenges, and resources during FRI management as well as emotional impact and socioeconomic consequences. No participant dropped out.

In total, 20 patients were willing to participate, at which point data saturation was reached based on data replication or recurrence in the content (Bowen, 2008). All interviews were audio recorded, anonymized, transcribed, and translated from German to English. Transcripts were returned to all included participants for comments. Here, no further corrections were made. To ensure the trustworthiness of this research (Nyirenda et al., 2020), credibility (internal validy) was enhanced by iterative questioning and rapport building. All participants were aware, that given statements will not be assigned to them personally and will have no effect regarding their professional position. Further, confirmability (objectivity) and reflexivity were given as the interviewer had no prior relationship with the participant and no personal or professional association with the inpatient ward. To enhance the confirmability, two researchers independently analyzed the data, which was then discussed by the whole research team.

Data were analyzed with content analysis. During the process, “Meaning units” were extracted from the transcripts, and condensed, i.e., shortening the text while preserving the essential part. Next, an abstraction was performed, meaning that the condensed units were labeled with a code by two of the researchers independently. After grouping the codes, data were categorized in themes as described previously (Graneheim and Lundman, 2004; Svensson et al., 2020; Walter et al., 2022b). Finally, themes were assigned to the categories, i.e., an interpretative meaning of related data. Codes were checked for consistency and validation and themes were discussed by the study team (Barry et al., 1999).

## Results

In total 20 participants were interviewed (Table 1).

**Table 1:** Demographic data of the study participants.

Participant	Sex	Age (years)	Locali-zation	ASA Score	CCI	Number of surgeries	Length of hospital stay (days)
Total *n* = 20	Female: *n* = 7 (35%); male: *n* = 13 (65%)	55.5 ± 14.6 (range: 18–79)	Shoulder: *n* = 3 (15%); tibia: *n* = 7 (35%); femur: *n* = 3 (15%); ankle: *n* = 5 (25%); foot: *n* = 2 (10%)	2.0 ± 0.9 (range: 0–4)	0.6 ± 0.8 (range: 0–2)	2.0 ± 1.55 (range: 1–7)	48.5 ± 32.0 (range: 10–126)
P1	Female	69	Shoulder	2	1	2	31
P2	Female	18	Tibia	1	0	2	18
P3	Male	59	Tibia	1	0	1	16
P4	Male	56	Femur	2	0	2	35
P5	Male	36	Shoulder	2	0	3	66
P6	Male	64	Ankle	3	2	1	28
P7	Female	79	Ankle	4	1	3	60
P8	Female	74	Tibia	2	1	7	108
P9	Female	71	Ankle	3	1	1	40
P10	Female	54	Foot	1	0	1	33
P11	Male	55	Ankle	1	0	2	24
P12	Male	52	Femur	2	0	1	22
P13	Male	62	Tibia	2	0	1	88
P14	Male	54	Femur	0	2	1	76
P15	Male	61	Shoulder	2	2	1	33
P16	Male	51	Foot	2	0	1	88
P17	Male	60	Tibia	3	0	1	33
P18	Male	64	Ankle	3	2	5	126
P19	Female	42	Tibia	3	0	3	34
P20	Male	29	Tibia	1	0	1	10

As an overarching category “the burden of FRI” was identified with the subthemes describing the challenges of the treatment in detail differentiated into „the emotional, mental and social aspects,” “socioeconomic consequences” and “resources” (Table 2). Patients described the practical challenges they experienced during their prolonged stay at the hospital as well as the challenges they faced at home.

**Table 2:** Summary of the main results emerged for each subtheme.

Subtheme	Main results
The emotional, and mental impact of FRI	Anxiety and fears even after a successful surgery
	Sleep disturbances
	Physical pain during and after recovery
	Side effects of the antibiotic therapy
	Restriction in daily activities, dependency on others
Socioeconomic consequences	Unemployment
	Job change
	Financial loss
	Reliance on social services
Resources	Spirituality and religion
	Yoga and meditation exercises
	Psychotherapy

### The emotional, and mental impact of FRI

The interviewed FRI patients still expressed anxiety and fears even after a successful surgery. „I have been afraid of every surgery” -P1. “So yes, I was afraid at the beginning that it would no longer close and that I would need such a skin transplant” -P2. The fear sometimes manifested although healing was adequate and there was no need for a renewed hospital visit. “I do not have blood drawn and I do not have an injection in my right arm. There is already something in the back of my head” -P1. This constant fear turned out to be very emotionally draining for patients. “Honestly, I felt very weak and somehow broken” -P2. While challenged by fear patients also reported physical pain during and after recovery. “I have had a lot of pain for a long time. Until the first of February, I had to take oxycodone, but it was not that I think about suicide”-P4. “There is always a dull pain, even if I do not move. Sometimes the two fractures rub against each other, and it goes clack clack and there is pain for seconds and it goes through the whole body. I feel it exactly at the fracture site when it will rain, and I also try to avoid painkillers.”- P15. In this stance, also side effects of antibiotic therapy were mentioned as a factor contributing to the suffering. In total, 18 out of 20 patients (90%) reported that they have struggled with massive intestinal problems. “I had severe pain. I took penicillin for 6 weeks, which I do not tolerate by nature, it was insane, the itching no one can imagine. The pain was terrible, my life was no longer worth living.”-P9. “What particularly affected me were the antibiotics. I was constantly nauseous and that has burdened me additionally. I had intense intestinal problems, six weeks of diarrhea and I could not eat anything”- P15. Many patients also mentioned having problems sleeping which added to the mental burden. “At the beginning I could not sleep so well because I was afraid that there was something there again or something like that”- P2. “Already extreme pain, I should try it, if possible, without opiates, because I have lost so much blood. I tried only with ibuprofen, but sleep was almost impossible, only half an hour, so hardly any sleep.”- P20.

Due to prolonged recovery, daily activities were restricted, and patients had to become dependent. “It was challenging when you can no longer do anything for yourself. Eating, washing, toileting, was terrible at first, but you grow into the situation, not overnight and you are grateful that someone is there for you and try to do as much as you can yourself.”- P14. This dependency was not easy to accept for most of the patients. “You feel like a 2nd class human being that you are no longer a full-fledged person.”-P15. “This has pulled me down brutally in the first moment because I had to start again from the beginning.”- P19. They had to accept that this situation does not only affect them but also their families and caregivers. “My family was hit very hard, because I could not do anything more”- P9. “A lot has changed for my daughter. My daughter had to drive me to every doctor’s appointment, she had to do the shopping and the cooking.”- P7. “I have a girlfriend and a child, and you want to do your job well and sometimes feel useless; already a burden to cope with it. I already reached the point where I no longer could handle it. At night-time I had so much pain that the next day was unbearable, it affected my mental state.”- P20. Additionally, almost all the patients interviewed expressed concerns for their future no matter how good or bad their recovery went. Main concern was the reappearance of an infection. “Yes, you often think that something could come again”- P7. “But the problem was still predominant and at times I just again had that fear. Hopefully there will not be any germs again”- P19. Also concerns about how life will change from now on were expressed. “Mentally I just once had a sag where it was said that they do not know if they can maintain my shoulder”-P5. “Psychologically, I was only burdened by the fact that I did not know how my career would continue.”- P20.

### Socioeconomic consequences

Further, during the interview some patients had a bigger loss of their formal social lives than others, but there were big changes throughout. “I lost my job. My husband is gone. Family life is destroyed. My children are now with my former husband.”- P19. A main change for patients was the current job situation. “So I was suspended and then I was unemployed. Since then, I had problems with finding a job. Let us put it this way because I still have these restrictions.”-P2. “Work was not so good and had to change the job because of it. It is now also not quite optimal and hope for another job with the same employer”- P20. While having a long recovery or restrictions maintaining a job is not easy and thus, financial problems many patients faced also create a negative impact on them. “But otherwise, financially it had a certain effect, because the salary is simply not as high as if I were working full time. I would say 20 percent less”-P3. Many patients also had to rely on social services or family members for finances which also had a negative effect on their mental health. “They have given me disability from the agricultural pension. I am entitled to 375 Euros, because I have not yet paid more. But they deduct 300 Euros monthly contributions. If this continues, I will not financially recover from it starting in June.”-P4.

### Resources

While interviewing patients and hearing about the difficulties the patients experienced it became clear that for some spirituality and religion were a positive coping strategy and resource. “Certain trust in God really helped me”-P1. “But in the meantime, it is definitely a hard situation and I think to myself that he still has something planned for me, the good Lord.”- P19. “I am catholic, but I am not into that, but still I prayed before the operation” -P15. “I have been doing yoga since I was 20 years old, this helped me a lot; meditation and positive thoughts” -P10. “I tried to keep my mental health positive, my physiotherapist helped me, I did some yoga to calm myself.” -P17.

## Discussion

In this study, FRI patients’ personal experiences and challenges were identified using a qualitative approach. Similar studies were carried out showing the negative impact on patients with a PJI (Moore et al., 2015; Palmer et al., 2020). This study focussed on FRI patients. The recovery experience may differ from patient to patient, but still all the participants reported a negative impact on distinct dimensions of their life.

### The emotional and mental impact of FRI

Due to not being able to know for certain if there is going to be a relapse of the infection patients expressed anxiety and fear. Restrictions in their mobility affected them physically and psychologically. Having to rely on family members also made changes in their social dynamics. With all these challenges patients got frustrated with many having depressive thoughts. Such mental health sequela are not uncommon after injuries and complications, which are associated with a high level of psychological distress (Johnson et al., 2019). For instance, it was shown that 20% of FRI patients reported moderate to severe symptom burden in an ICD-10 chapter F symptom rating, even years after successful treatment including infect eradication and stable bone consolidation (Walter et al., 2021a). This finding is also in line with a previous qualitative analysis, in which patients described their challenge to cope with the loss of physical function and mobility. Some even reported depression and suicidal thoughts as a consequence of the sense of disablement (Moore et al., 2015). In addition, it is well-known that depression in patients with chronic medical illness enhances symptom burden which leads to additive function impairment (Katon and Ciechanowski, 2002; Iakovou and Schulpis, 2019). Further, a positive correlation between depression, anxiety, and the severity of pain exists (Pan et al., 2019). While research on the effect of psychological interventions in this population is scarce, these findings highlight the need to integrate psychological support in daily clinical care for patients with implant-associated infections (Kunutsor et al., 2017). For instance, weekly group sessions incorporating easily applicable methods such as functional relaxation or breathing exercises could be beneficial in improving the patients’ quality of life (Zaccaro et al., 2018; Walter et al., 2022a).

Due to the frustration, some patients seemed to show insufficient compliance regarding the treatment. Also, in the field of PJI it was reported that non-compliance often resulted from the fact that patients were not aware of the severity of the infectious complication and thus, did not expect a long-time course of treatment (Walter et al., 2022b). To address this uncertainty among patients more in-depth information could be given to them and patient education programs could be launched. Here, evidence exists from studies with osteoarthritis patients showing that patients with the highest knowledge of their condition also showed better coping, less depressive symptoms, and less pain (Axford et al., 2008).

### Socioeconomic consequences

One major issue reported by the patients was the challenge of maintaining a job or finding a new one with mobility restrictions. Many patients were not capable of looking for a job or going back to their old jobs, having new living situations, or having people take care of them. All patients were fearful about their finances and how they can sustain themselves, their families, or their houses. A recent qualitative study from Moore et al. focussing on PJI patients also concluded that the treatment has a massive impact on family and personal relationships (Moore et al., 2015). A recent publication showed that postoperative infections after fractures of the extremities or pelvis were associated with a 45% increase in the odds of receiving Social Security benefits (odds ratio, 1.45; 95% CI, 1.25–1.68; *p* < 0.001) (O'Hara et al., 2021). This aspect should be communicated to the patients and adequate resources should be allocated to support services, including mental health and social care. Additionally, also in turn, socially deprived patients are at higher risk for revision surgeries due to infection as well as amputation as shown for ankle fractures (Wolfstadt et al., 2019).

### Resources

Some patients reported that spirituality was a positive resource on their way to recovery. Spiritual beliefs are an essential factor for enhancing mental health, easing up depression and anxiety, as well as providing meaning and purpose, especially during times of suffering (Koenig, 2010). It has been shown that spirituality is directly correlated with psychological well-being (Bożek et al., 2020), and thus, the management team should be open to it. Also, independent of any spiritual beliefs, yoga, and meditation was reported as beneficial by the patients for calming themselves. Positive effects of mind–body interventions, and especially mindfulness meditations are widely reported for a range of diseases (Sampaio et al., 2017). While the effect of such an adjunct therapy approach has still to be evidenced in FRI patients (Walter et al., 2022a), improvements in functional outcomes were reported in patients undergoing primary arthroplasty procedures after relaxation exercise (Lim et al., 2014; Eymir et al., 2022).

### Limitations

A limitation of this study is that only the experiences of FRI patients in one clinic, specialized in bone and joint infection were captured. Thus, the generalizability of the results may be limited, and findings may slightly differ when transferred to centres with less expertise in the management of FRI as well as to the healthcare in other countries. In addition, the time of the interviews was relatively short (30 to 46 min). However, at the end each participant was asked whether there is anything else they would to add, or anything they wish to talk about that we have not covered already, which was denied by all participants.

### Practical applications and future prospects

The findings of this study highlight the patients’ need for psychological support and advocate for integrating psychological interventions into clinical practice. Here, a wide range of interventions is possible including for instance relaxation, cognitive behavioral therapy, hypnosis, guided imagery, or emotional counseling. However, it has to be noticed, that such an approach is still in its infancy and a gap in the literature exists, also when it comes to primary arthroplasty without the development of an infection (Bay et al., 2018). Therefore, the effectiveness of psychological interventions in improving patient-reported outcomes still has to be explored. In the same stance, it is unclear which kind of intervention, mode of delivery, and timing would be optimal for the considered patient cohort. Hence, randomized-controlled trials to address this aspect are urgently required. Further, while in other disciplines such as oncology, tumor boards are well-established and proven beneficial, the role of multidisciplinary teams in bone and joint infection management has only recently been scientifically looked at (Rupp et al., 2023). The presented results encourage collaborative approaches and deem the presence of a psychologist as part of the treatment team as warranted.

## Conclusion

In conclusion, this study underlines the negative impact of FRI on the emotional and mental levels experienced from the patients’ perspective highlighting their personal challenges as well as resources during the recovery process. Not being well informed about possible negative outcomes or restrictions makes it harder for patients to accept the situation and patients expressed a need for better information and certainty. Acknowledging these aspects could improve clinician-patient communication and multiple understanding. Further, patients developed constant anxiety and other psychological disturbances. This finding indicates the potential of integrating psychological services as well as adjunct mind–body therapies in the treatment of FRI. Whereas multidisciplinary approaches are warranted and recommended in the literature, opportunities to enhance not only patients’ somatic but also their mental state are still underestimated.

## Data availability statement

The raw data supporting the conclusions of this article will be made available by the authors, without undue reservation.

## Ethics statement

The studies involving human participants were reviewed and approved by the institutional ethics committee of the University Hospital Regensburg (file number 21-2464-101). The patients/participants provided their written informed consent to participate in this study.

## Author contributions

BW, MR, VA, and NW conceptualized the work. BW and NW acquired the data. NW, BW, and MR analysed the data. BW, MR, VA, and NW interpreted the data. BW and NW drafted the manuscript. BW, MR, VA, and NW revised it critically for important intellectual content and approved the version to be published. All authors contributed to the article and approved the submitted version.

## Conflict of interest

The authors declare that the research was conducted in the absence of any commercial or financial relationships that could be construed as a potential conflict of interest.

## Publisher’s note

All claims expressed in this article are solely those of the authors and do not necessarily represent those of their affiliated organizations, or those of the publisher, the editors and the reviewers. Any product that may be evaluated in this article, or claim that may be made by its manufacturer, is not guaranteed or endorsed by the publisher.
